# Antisense Transcription Regulates the Expression of the Enterohemorrhagic *Escherichia coli* Virulence Regulatory Gene *ler* in Response to the Intracellular Iron Concentration

**DOI:** 10.1371/journal.pone.0101582

**Published:** 2014-07-09

**Authors:** Toru Tobe, Hilo Yen, Hiroki Takahashi, Yoko Kagayama, Naotake Ogasawara, Taku Oshima

**Affiliations:** 1 Department of Biomedical Informatics, Graduate School of Medicine, Osaka University, Suita, Osaka, Japan; 2 Department of Microbiology and Immunology, Graduate School of Medicine, Osaka University, Suita, Osaka, Japan; 3 Medical Mycology Research Center, Chiba University, Chiba, Chiba, Japan; 4 Graduate School of Information Science, Nara Institute of Science and Technology, Ikoma, Nara, Japan; University of Massachusetts Medical School, United States of America

## Abstract

Enteric pathogens, such as enterohemorrhagic *E. coli* (EHEC) O157:H7, encounter varying concentrations of iron during their life cycle. In the gastrointestinal tract, the amount of available free iron is limited because of absorption by host factors. EHEC and other enteric pathogens have developed sophisticated iron-responsive systems to utilize limited iron resources, and these systems are primarily regulated by the Fur repressor protein. The iron concentration could be a signal that controls gene expression in the intestines. In this study, we explored the role of iron in LEE (locus for enterocyte effacement) virulence gene expression in EHEC. In contrast to the expression of Fur-regulated genes, the expression of *LEE* genes was greatly reduced in *fur* mutants irrespective of the iron concentration. The expression of the *ler* gene, the LEE-encoded master regulator, was affected at a post-transcription step by *fur* mutation. Further analysis showed that the loss of Fur affected the translation of the *ler* gene by increasing the intracellular concentration of free iron, and the transcription of the antisense strand was necessary for regulation. The results indicate that *LEE* gene expression is closely linked to the control of intracellular free iron homeostasis.

## Introduction

Enteric pathogens that infect the mammalian gut use specific traits, referred to as virulence factors, to grow on the intestinal surface and sometimes pass through the epithelial barrier to reach deeper tissues. To activate the expression of virulence factors at the appropriate time and to target niches, pathogens often sense the chemical and/or physical conditions of the intestinal environment. The intestines contain a variety of environmental factors that are altered by ingested food, the metabolic activity of the microflora, the location in the intestine and the physiological conditions of the host. One or several of these factors are thought to be signal(s) that regulate the expression of virulence genes by enteropathogens.

Iron is essential for living systems but is toxic at high levels. Many enzymes require iron as a cofactor for catalytic activity [1]. However, free iron accelerates the production of hydroxyl radicals by the Fenton reaction [2]. Bacteria possess several specific systems to capture and take up iron, and the associated genes are strictly regulated by the iron concentration through an iron-interacting DNA binding protein [3]. Fur, the ferric uptake regulator, is a repressor of genes involved in iron utilization [4]. At low concentrations of iron, Fur not bound by iron (apo-Fur) is released from the operators of target genes, and the promoters become active. In addition to iron utilizing systems, Fur and iron regulate a variety of genes involved in respiration, acid resistance, oxidative stress responses, and virulence [5]. The expression of virulence genes is known to be regulated by the iron concentration in a variety of enteric bacteria, such as *Vibrio* spp, *Pseudomonas aeruginosa*, *Yersinia* spp, *Salmonella* spp, and pathogenic *Escherichia coli* [6]. In enterohemorrhagic *E. coli* (EHEC), stx (Shiga-toxin) genes are directly regulated by Fur, and their expression is activated when the iron concentration decreases [7], [8].

Enterohemorrhagic *E. coli* (EHEC) are human pathogens that cause a wide range of symptoms from watery diarrhea to bloody diarrhea and hemolytic uremic syndrome (HUS) [9]. Colonization of the intestinal mucosa by EHEC causes lesions known as A/E lesions, which are characterized by intimate attachment of the bacteria to the host cell surface and the disruption of the brush borders [10]. The virulence factors necessary for A/E lesion induction are primarily encoded by the chromosomal region called LEE (locus for enterocyte effacement) [11], [12]. LEE also encodes the transcriptional regulator Ler, which activates the transcription of other *LEE* genes. The expression of *LEE* genes is regulated in response to changes in the environmental conditions, and some responses are regulated at the *LEE1* promoter, which is a promoter in the *LEE1* operon, which contains the *ler* gene [13]. In EHEC, one of the regulatory proteins necessary for the activation of the *LEE1* promoter is Pch, which is encoded by *pch* genes at other chromosomal loci [14], [15]. Both Ler and Pch coordinate the regulation of virulence genes, including *LEE* genes and non-LEE effector genes, in response to changes in environmental conditions [13], and play important roles in integrating the virulence regulon into the backbone regulatory systems in *E. coli* [16]–[19].

In this study, we investigated the response of *LEE* genes to changes in the iron concentration and the role of the Fur regulator in *LEE* gene expression. To elucidate the mechanism of Fur-dependent expression, we also examined how bacteria with different genetic backgrounds respond to changes in the intracellular free iron concentration. Finally, we identified a novel regulatory mechanism mediated by the transcription of the anti-sense strand.

## Results

### Repression of LEE-encoded virulence factor production by iron

**Figure 1:**
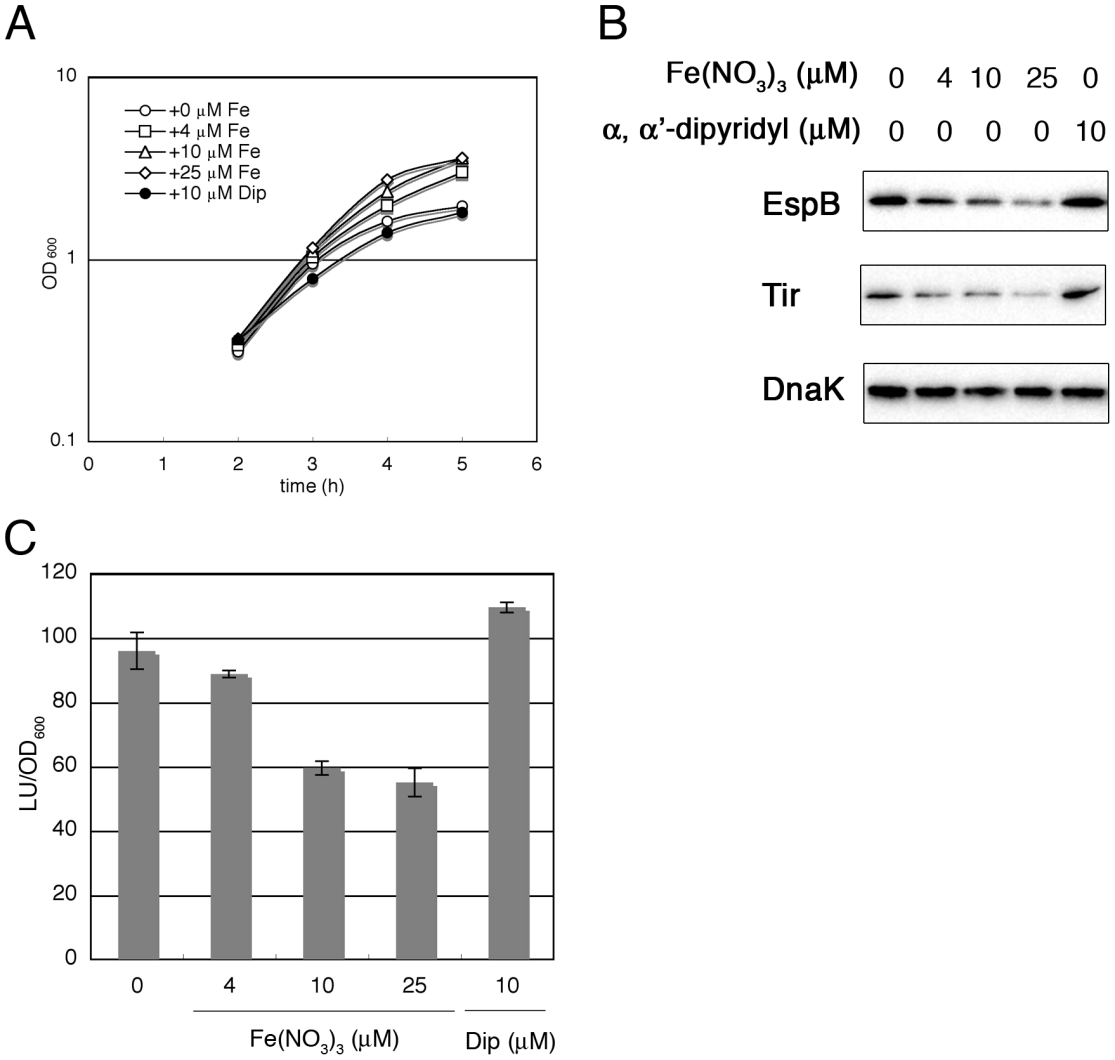
Effect of a high concentration of iron on the growth and expression of LEE-encoded virulence factors. EHEC O157 Sakai was grown in DMEM containing an additional Fe(NO_3_)_3_ or α,α′-dipyridyl (Dip). A. Effect of various concentrations of iron in the medium on the growth of wild-type EHEC O157 Sakai. The growth of the cultures was monitored by measuring the OD_600_. B. Effect of various concentrations of iron on the expression levels of EspB and Tir. Proteins in whole lysates were detected by immunoblotting with specific antibodies. C. Effect of various concentrations of iron on *LEE1* promoter activity. The *LEE1* promoter activity in wild-type EHEC O157 Sakai in the exponential phase of growth was monitored using the P*_LEE1_*-*lux* operon fusion.

EHEC infection targets the intestinal mucosa, where there is little free iron. The expression of virulence factors involved in adherence and colonization is activated during bacterial growth in intestinal environment. A low concentration of iron could be an environmental signal that affects the expression of virulence genes in EHEC. To test this hypothesis, we compared the production of LEE-encoded virulence factors between cultures grown in medium containing various concentrations of iron. The expression of *LEE* genes was elevated in EHEC grown in Dulbecco's modified Eagle medium (DMEM), which contains only 0.25 µM Fe(NO_3_)_3_, compared with that in bacteria grown in LB medium, which contains ∼7.6 µM Fe [20]. Increasing the Fe(NO_3_)_3_ concentration from 4 to 25 µM in DMEM did not inhibit the growth of EHEC and instead stimulated growth, resulting in a higher density in the stationary phase (Fig. 1A). The addition of iron to DMEM decreased the production of LEE-encoded proteins (Fig. 1B). The levels of EspB and Tir were reduced compared with those in bacteria grown in unmodified DMEM, which contains a relatively low amount of Fe(NO_3_)_3_. To further examine the effect of the iron concentration on LEE-encoded protein production, iron was depleted from DMEM by adding an iron-specific chelator, α,α′-dipyridyl (Dip), and the production of EspB in EHEC was examined. As shown in Fig. 1A and 1B, the depletion of iron from DMEM by adding α,α′-dipyridyl slightly increased the levels of EspB and Tir produced in EHEC. These results strongly suggest that the expression of *LEE* genes is affected by the iron content of the medium.

The production of both EspB and Tir was affected by changing the iron concentration in the medium, suggesting that the transcription of the regulatory gene in LEE, *ler*, could be affected. Ler is the first gene in the *LEE1* operon. The promoter activity of the *LEE1* operon was monitored using fusions with the *lux* operon of *Photorhabdus luminescens*. The promoter activity was decreased with increasing iron concentration in the medium (Fig. 1C). When an iron chelator was added in the medium, the promoter activity was increased compared with that in bacteria grown in the unmodified medium (Fig. 1C). These results are consistent with the above results for the production of LEE-encoded proteins and suggest that the expression of all *LEE* genes is affected by the iron concentration in the medium.

### Fur is required for full expression of *LEE* genes

**Figure 2:**
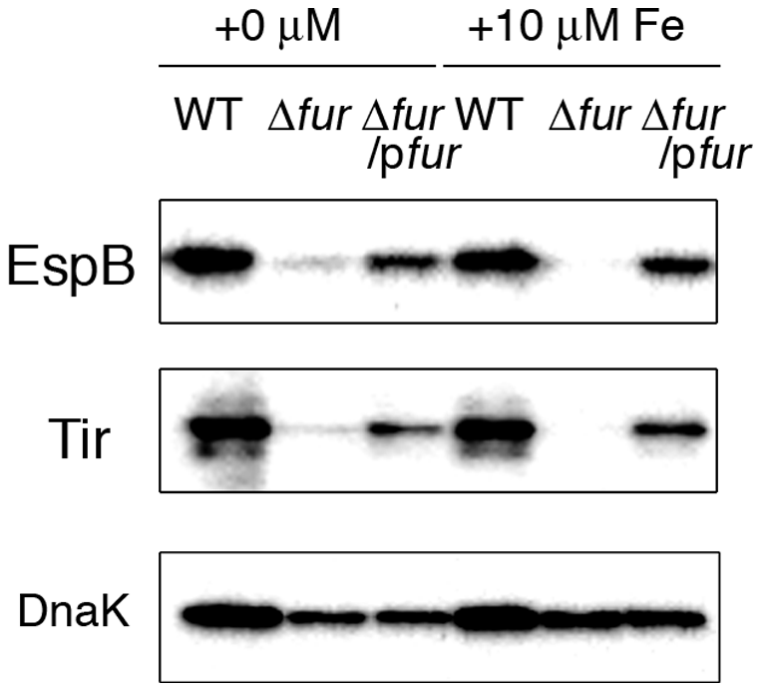
Expression of LEE-encoded virulence factors in the *fur* mutant. Wild-type (WT), fur mutant (Δ*fur*) and the complemented strain (Δ*fur*/p*fur*) of EHEC O157 Sakai were grown in DMEM with/without 10 µM Fe(NO_3_)_3_ to the logarithmic growth phase. EspB and Tir were detected by immunoblotting.

The ferric uptake regulator Fur is a key regulator of iron metabolism [21]. In the presence of iron at relatively high concentrations, Fe^2+^-bound Fur binds as a dimer to iron-responsive promoter regions and represses promoter activity [4]. In contrast, under low-iron conditions, Fe^2+^-free Fur is released from these binding sites, and gene expression is de-repressed. To examine the involvement of Fur in the iron-responsive regulation of *LEE* genes, a *fur*-deletion mutant of EHEC was created, and the production level of the LEE-encoded protein EspB was compared with that in the parental strain during growth in DMEM containing a low (0.25 µM) or high (10.25 µM) concentration of Fe(NO_3_)_3_. As shown in Fig. 2, the deletion of the *fur* gene reduced the production of EspB under both the low and high iron conditions. Even in DMEM without added iron, the level of EspB was remarkably reduced in the *fur* mutant compared with the level in the wild type. The introduction of the *fur* gene on a multi-copy number plasmid restored the production of EspB in the mutant under both conditions (Fig. 2).

**Figure 3:**
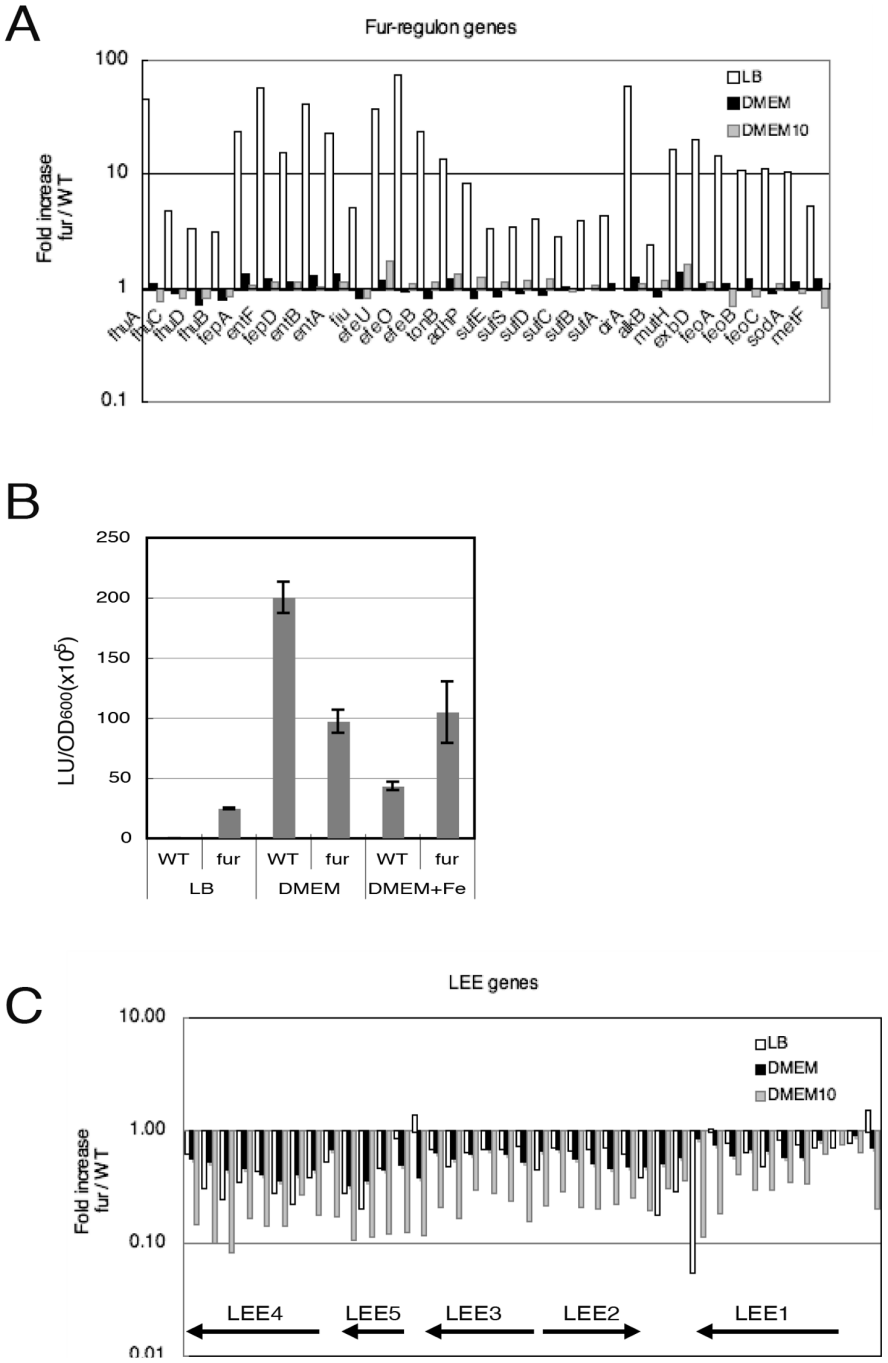
Responses of Fur regulon genes and *LEE* genes. Transcriptomic analysis was performed with wild-type and the *fur* mutant EHEC O157 Sakai grown in LB or DMEM with/without 10 µM Fe(NO_3_)_3_, and the ratio of the transcript level for each gene in the *fur* mutant to the transcript level in the wild type was obtained. A. Ratios of the transcript levels of genes in the Fur regulon. B. Promoter activity of the *fepA* gene in wild-type and *fur* mutant bacteria grown in LB or DMEM with/without 10 µM Fe(NO_3_)_3_. The promoter activity was measured using a luciferase-operon fusion. C. Ratios of the transcript levels of *LEE* genes in EHEC grown in LB or DMEM with/without 10 µM Fe(NO_3_)_3_. The ratios were obtained from the transcriptome data.

Because the concentration of iron in DMEM is low, it is unlikely that Fur represses genes belonging to the Fur regulon [5]. Using GeneChip, the transcript level of each gene was compared between the wild-type and *fur* mutant EHEC. In EHEC grown in DMEM, there were no significant differences in the transcription levels of genes of the Fur regulon between the wild type and the *fur* mutant (Fig. 3A). This result indicates that the genes of the Fur regulon in wild type EHEC are de-repressed and fully expressed when the bacteria are grown in DMEM. To confirm the response of Fur-regulated promoters, the promoter activity of the *fepA* gene was measured using an operon fusion with the luciferase gene. As expected, the promoter activity in wild-type EHEC was much higher when grown in DMEM than when grown in LB, and the activity was not further enhanced in the *fur* mutant (Fig. 3B). In contrast, in LB, which contains approximately 7.6 µM iron, the transcript levels of genes of the Fur regulon were markedly increased in the *fur* mutant compared with those in the wild type. This result indicates that the Fur regulon genes are repressed by Fur, as observed in *E. coli* K-12 when grown in LB medium. The transcription of Fur regulon genes in wild-type EHEC was not fully repressed in DMEM containing an additional 10 µM of Fe (final concentration at 10.25 µM) (Fig. 3A). The *fepA* promoter activity in EHEC grown in DMEM containing an additional 10 µM of Fe was repressed by Fur but was higher than that of bacteria grown in LB (Fig. 3B). These results indicate that the addition of 10 µM Fe was not sufficient to fully repress Fur-regulated genes in EHEC grown in DMEM, which may contain components with iron-chelator activity.

In contrast, the transcription levels of *LEE* genes in the *fur* mutant were reduced compared with those in the wild type when grown in DMEM or DMEM plus 10 µM Fe (Fig. 3C). A decrease in the transcript levels in the *fur* mutant was also observed when EHEC was grown in LB (Fig. 3C). These results are consistent with the observations for EspB protein production, which was decreased in the *fur* mutant grown in both iron-poor and iron-rich medium. Therefore, it is likely that Fur can affect *LEE* gene expression even at low concentrations of iron, at which Fur is unable to repress the expression of genes of the Fur regulon, in a manner independent of its repressor activity.

### Fur regulates the expression of genes that regulate *LEE* gene expression

**Figure 4:**
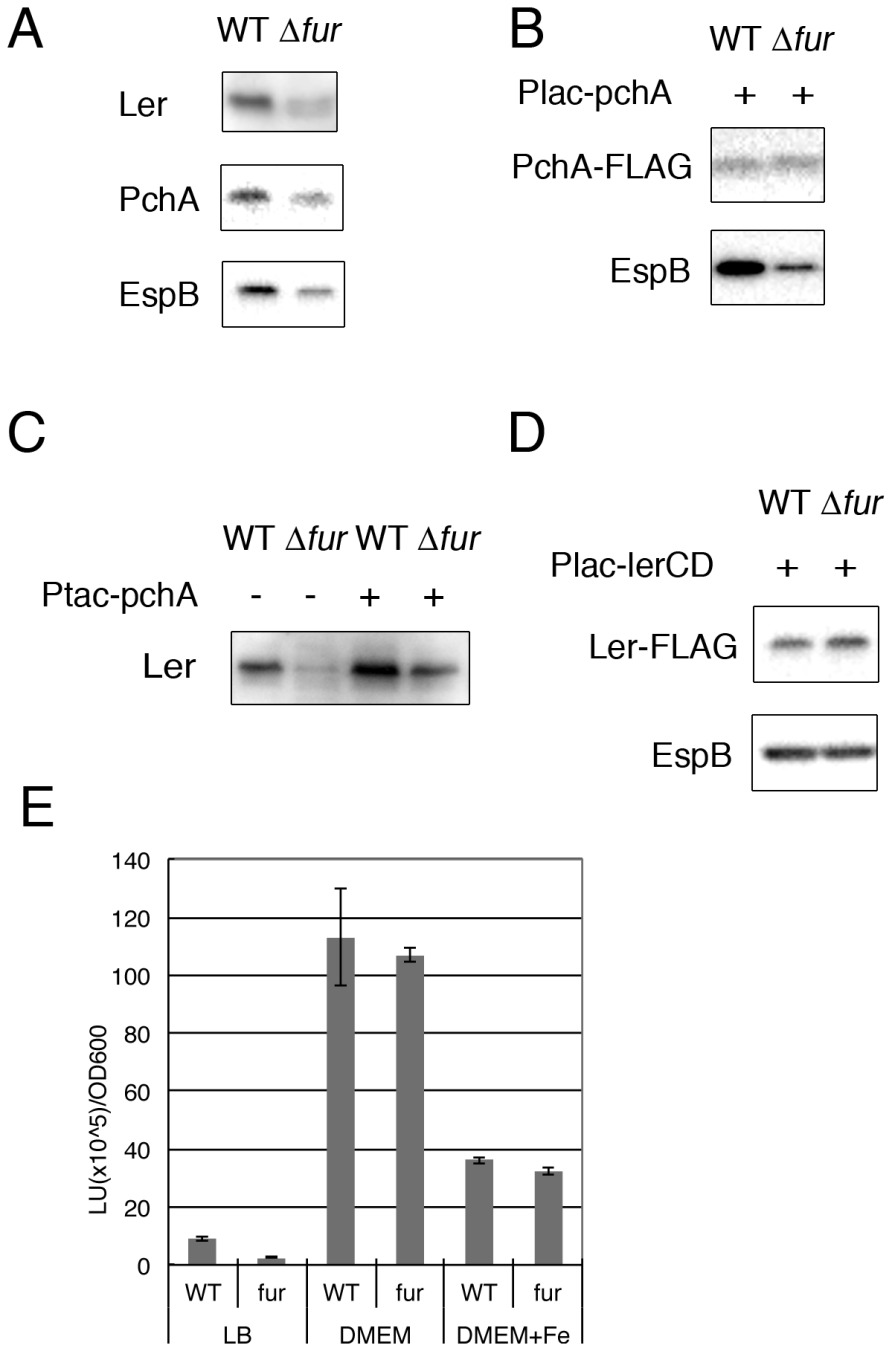
Expression of regulators of *LEE* genes in the *fur* mutant of EHEC. A. Ler, PchA and EspB expression in the *fur* mutant. EHEC O157 Sakai *pchA*-FLAG or the corresponding *fur* mutant was grown in DMEM. Ler and EspB were detected using specific antibodies, and PchA was detected using an anti-FLAG antibody. B. The effect of constitutive expression of PchA in the *fur* mutant. Wild-type or *fur* mutant EHEC O157 Sakai harboring P*_lac_*-*pchA*-FLAG was grown in DMEM containing IPTG (2 µM). PchA-FLAG and EspB were detected using anti-FLAG and anti-EspB antibodies, respectively. C. Effect of PchA overexpression on *ler* expression in the *fur* mutant. The EHEC O157 Sakai *ler*-FLAG strain and the corresponding *fur* mutant harboring the P*_tac_*-*pchA* plasmid were grown in DMEM containing IPTG (5 µM). Ler-FLAG was detected using an anti-FLAG antibody. D. Rescue of EspB expression in the *fur* mutant by the P*_lac_*-*ler* plasmid. EHEC Δ*ler* and Δ*ler*Δ*fur* mutants harboring the plasmid carrying P*_lac_*-*ler*CD were grown in DMEM containing IPTG (5 µM). E. Promoter activity of the *LEE1* operon in the *fur* mutant. Wild-type or *fur* mutant EHEC O157 Sakai harboring pLux-P*_LEE1_* were grown in LB or DMEM with/without 10 µM Fe(NO_3_)_3_. The promoter activity was measured using the luciferase activity (LU/OD_600_). The averages of three independent experiments are shown with the SEM.

Because expression of *LEE* genes is regulated coordinately at the transcription step, Fur could affect the production of regulator(s) of *LEE* genes, such as Ler and Pch. To elucidate the involvement of Fur in the expression of the *ler* and *pch* genes, an EHEC strain possessing a FLAG-tagged *pchA* gene but neither *pchB* nor *pchC* was compared with the isogenic *fur* mutant. The bacteria were grown in DMEM without any additional iron, and Ler, PchA-FLAG and EspB were detected by immunoblotting using specific antibodies against each protein. As shown in Fig. 4A, the amount of EspB protein in the *fur* mutant was reduced compared with that in the parental strain. The production of both regulators, Ler and PchA, was also markedly reduced in the *fur* mutant (Fig. 4A). Because the promoter of the *LEE1* operon, which includes the *ler* gene, is positively regulated by PchA, the decreased expression of the *pch* gene in the *fur* mutant could be a cause of the reduced expression of Ler and the *LEE* genes. To test this hypothesis, we examined the expression of *LEE* genes in EHEC expressing PchA constitutively from the P*_lac_*-*pchA*-FLAG gene. When PchA-FLAG was expressed in the *pch* mutant of EHEC, EspB protein was produced at a level comparable to that in the wild type. In the *fur* mutant, the EspB expression level was much lower than that in the wild type, even though the same level of PchA protein was expressed (Fig. 4B). Furthermore, the expression of *ler* was monitored in EHEC possessing the *ler*-FLAG gene and the P*_lac_*-*pchA* gene. Although the amounts of Ler in both strains were increased by the expression of *pchA* from the *lac* promoter, the level in the wild type was much higher than that in the *fur* mutant (Fig. 4C).

Next, *ler* with the *lac* promoter (P*_lac_*-*ler*-FLAG) was introduced into the *fur* mutant, and the effect of the constitutive expression of Ler was examined. When Ler was produced in the *fur* mutant at the same level as in the wild type, the expression of EspB in the *fur* mutant reached the same level as that in the wild type (Fig. 4D). This result suggests that the *fur* mutation did not affect the activity of the Ler protein but did affect the expression of the *ler* gene. Next, we examined the promoter activity of the *LEE1* operon in the *fur* mutant using the P*_LEE1_*-luciferase fusion (Fig. 4D). As shown in the previous experiment, the promoter activity was reduced in DMEM containing an additional 10 µM of Fe(NO_3_)_3_ and in LB compared with the activity in unmodified DMEM, whereas, the promoter activity in the *fur* mutant was the same as that in the wild type irrespective of the iron concentration. These results clearly indicate that Fur is not necessary for the activation of the *LEE1* promoter and that the reduction in the promoter activity at high iron concentrations is not the mechanism of Fur-mediated regulation.

### The sequence downstream of the *ler* gene is necessary for Fur-dependent expression

**Figure 5:**
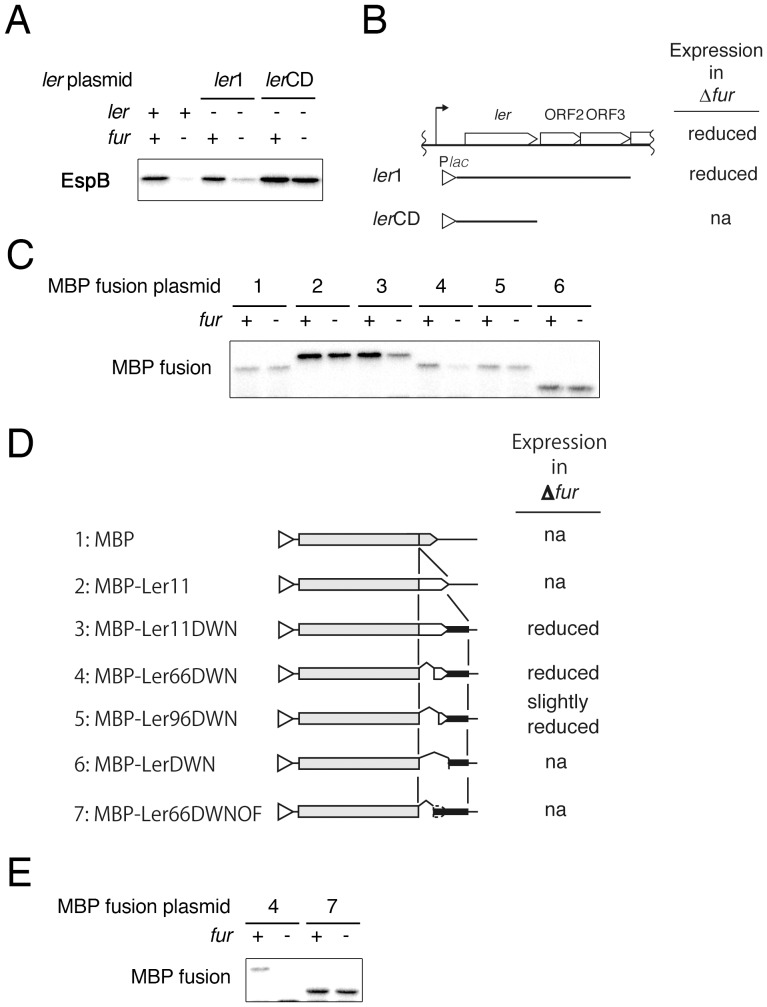
Necessity of the *ler* downstream sequence for Fur-dependent expression. A. Expression of EspB in a *fur* mutant harboring P*_lac_*-*ler* with or without the downstream sequence. P*_lac_*-*ler* fusion with (*ler*1) or without (*ler*CD) the downstream sequence was introduced into the EHEC Sakai Δ*ler* or Δ*ler*Δ*fur* mutant. The expression of EspB was detected by immunoblotting using an anti-EspB antibody. B. Structure of the P*_lac_*-*ler* fusions used in A and the EspB expression levels. C. Production of MBP-Ler fusion proteins in the *fur* mutant. Plasmids carrying a variety of MBP-Ler fusion constructs were introduced into the EHEC Δ*ler* and Δ*ler*Δ*fur* mutants. MBP and MBP-Ler fusion proteins were detected by immunoblotting using an anti-MBP antibody. D. Structure of the MBP-Ler fusion genes used and the levels of fusion protein production. Gray boxes and white boxes represent MBP and part of the Ler protein, respectively. Thick lines indicate the downstream sequence of *ler*. Open triangles indicate the *tac* promoter. E. Production of MBP fusions with in-frame or out-of frame fusion. Plasmids carrying MBP with the *ler* downstream sequence connected in-frame or out-of frame were introduced into the EHEC Δ*ler* and Δ*ler* Δ*fur* mutants.

Because the promoter of the *LEE1* operon was not affected by Fur, post-transcriptional regulation could be the target of the Fur-dependent expression of *LEE* genes. Furthermore, when the *ler* gene with only the coding sequence was expressed from the *lac* promoter, EspB production became independent of Fur, suggesting that the sequence around the *ler* gene is necessary for Fur-dependent expression. To test the hypothesis, a plasmid carrying P*_lac_*-*ler* with the downstream sequence was introduced into the *ler* mutant and *ler fur* double mutant, and the expression levels of EspB were compared. In EHEC expressing Ler from *ler* with the downstream sequence, the expression of EspB was reduced by the presence of the *fur* mutation, whereas the same level of expression as that in the *fur*-positive strain was observed in the *fur* mutant when the protein was expressed from the *ler* gene without the downstream sequence (Fig. 5A and B). To further explore the role of the *ler* downstream sequence in *ler* expression, the expression levels of MBP fusion proteins from various MBP-*ler* fusion constructs were examined in the *fur* mutant. When MBP was fused with the *ler* C-terminus without the downstream sequence, the expression of the MBP-Ler fusion protein was not affected by the *fur* mutation (MBP fusion plasmid 2: MBP-Ler11). In contrast, the expression of the same MBP-Ler fusion protein from the MBP-Ler fusion construct with the additional downstream sequence was reduced by the *fur* mutation (Fig. 5C and D, plasmid 3: MBP-Ler11DWN). Next, we attempted to identify the region necessary for Fur-dependent expression. The deletion of the N-terminal 198 bp (from ATG) of the *ler* coding sequence had no effect on reduced expression, but the deletion of 1-288 bp of the coding sequence resulted in the expression of the MBP-Ler fusion protein in the *fur* mutant at almost the same level as in the *fur*-positive strain (Fig. 5C and D, compare plasmids 4 and 5). Furthermore, to explore the necessity of the translation of the *ler* portion of the fusion, a stop codon was placed at the junction of the MBP and *ler* coding sequences in plasmid 4. The resulting plasmid lost *fur*-dependent expression (Fig. 5D and E, plasmid 7). These results suggest that at least two sequence regions, the sequence from 198 to 288 relative to the ATG of *ler* and the 233 bp downstream of the stop codon of *ler*, are involved in the Fur-dependent expression of *ler*. Furthermore, the translation of bp 198–288 relative to the ATG of *ler* was markedly affected by the absence of the *fur* gene.

### Post-transcriptional regulation of *ler* by antisense RNA

**Figure 6:**
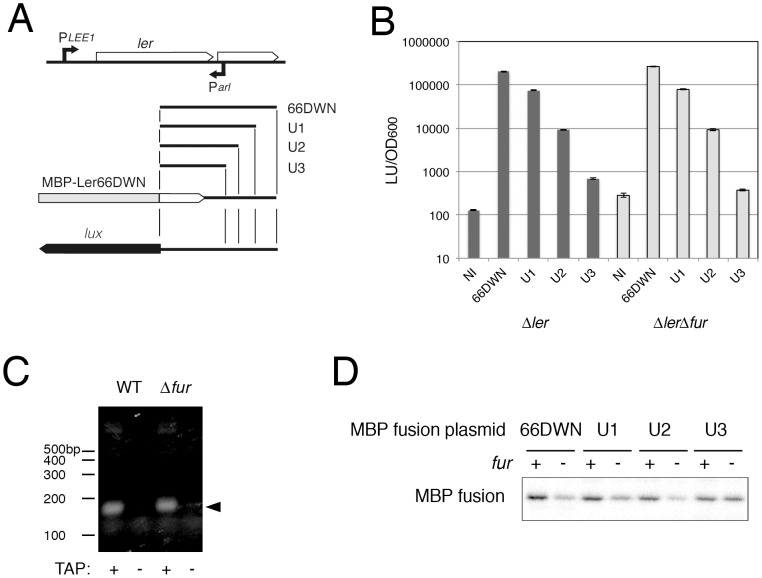
Role of transcription from the antisense strand in *ler* expression. Deletion derivatives of the MBP-*ler*66DWN fusion gene or luciferase operon fusions were created by inserting fragments with various deletions (U1, U2 and U3) (A). B. Promoter activity from the opposite strand. pLux-*ler*66DWN and deletion derivatives were introduced into the EHEC Sakai Δ*ler* and Δ*ler*Δ*fur* mutant, and the promoter activity was measured using the luciferase activity. C. Production of arl RNA. 5′ end of *arl* RNA was amplified by 5′ RACE method. Arrowhead indicates PCR products. D. Production of MBP-Ler66DWN fusion proteins from the deletion derivatives. Plasmids expressing MBP-Ler66DWN fusions with various lengths of the downstream fragment were introduced into the Δ*ler* and Δ*ler*Δ*fur* mutants, and the production of the MBP-Ler66DWN fusion was detected by immunoblotting with anti-MBP.

One possible mechanism for the regulation of the translation of *ler* is competition for translational processing by transcripts from the opposite strand. Because the downstream sequence of *ler* is necessary for the observed regulation, we assessed the promoter activity in this region. The DNA fragment spanning from +620 (from ATG of *ler* gene) to +198 was placed upstream of the luciferase operon, and the luciferase activity in wild-type and *fur* mutant EHEC O157 was quantified. Promoter activity from the opposite direction was detected. To explore role of transcription from the opposite strand in the *fur*-dependent expression of the *ler* gene, deletion derivatives of the downstream region (upstream of the opposite strand) were fused to create MBP-Ler gene fusions or luciferase operon fusions (Fig. 6, A). The promoter activity was decreased by shortening the fragment and by the deletion of 192 bp (U3) (Fig. 6, B). To confirm the transcript from opposite strand, the 5′-end specific sequence was amplified with 5′ RACE method (see Materials and Methods) using a primer for the opposite strand in the *ler* coding region. A single band around 180 bp was detected from TAP (Tabacco acid pyrophosphatase)-treated RNA of wild type or *fur* mutant, but not from TAP-untreated RNA (Fig. 6, C). Therefore, the transcript from the opposite promoter is designated the *arl* RNA (antisense regulator of *ler* RNA). The production of MBP-Ler fusions from the plasmids 66DWN, U1 and U2 was reduced in the *fur* mutant, whereas MBP-Ler production from U3 was not affected by the *fur* mutation (Fig. 6, D). The promoter activities were almost the same in *fur*-positive strains and the *fur*-negative strain, indicating that the promoter activity for the *arl* RNA was not affected by the *fur* mutation. These results indicate that the transcription of the *arl* RNA on the opposite strand is necessary for the reduction in Ler production caused by the *fur* mutation.

### Intracellular iron is involved in the repression of LEE expression

**Figure 7:**
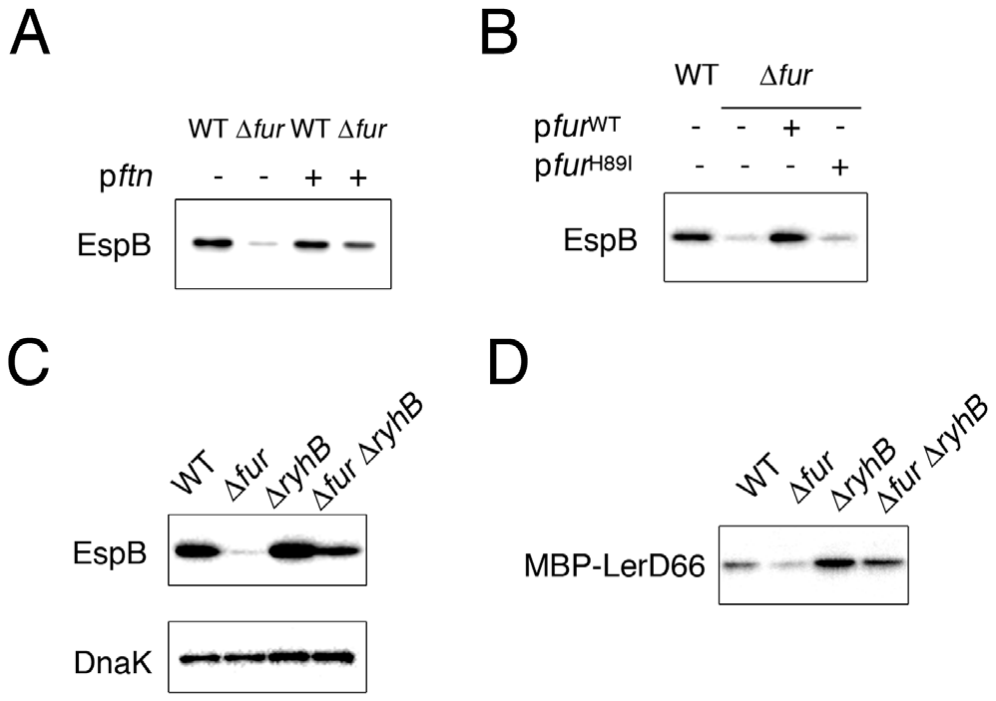
Effect of iron binding proteins and *ryhB* mutation on Fur-dependent expression. A. Effect of the overexpression of the ferritin gene. Wild-type or *fur* mutant EHEC harboring pGEM-*ftnA* was grown in DMEM. EspB was detected by immunoblotting using an anti-EspB antibody. B. Role of the iron-binding capacity of Fur. The *fur* mutant expressing wild-type Fur or a Fur (H89I) mutant was grown in DMEM. C. Effect of *ryhB* mutation on Fur-dependent expression. The EspB expression levels in wild-type, *fur* mutant, *ryhB* mutant, and *fur ryhB* mutant EHEC were monitored by immunoblotting using an anti-EspB antibody. D. Effect of *ryhB* on the expression of MBP-Ler fusion proteins. Wild-type or mutant EHEC harboring plasmid encoding MBP-LerD66DWN was grown in DMEM, and the production of MBP-Ler fusion proteins was detected by immunoblotting using an anti-MBP antibody.

The iron ion concentration in DMEM is low, and at this concentration, Fur does not bind to its target sites and Fur regulon genes are expressed, as shown above. It is unlikely that the binding of Fur to chromosomal targets directly activates *pch* promoters or the *LEE1* promoter. Furthermore, the binding of Fur to the promoter regions of *pch* genes and the *LEE1* operon was not observed even in media containing high concentrations of iron, such as LB; such binding was also absent in DMEM (data not shown). Because a high concentration of iron in the medium decreases the expression of *LEE* genes and because the *fur* mutation causes a reduction in the production of the cytoplasmic iron-binding protein ferritin, we hypothesized that intracellular free iron ions could suppress the expression of *LEE* genes. To test this hypothesis, we expressed the iron-binding protein ferritin in EHEC and compared the production of LEE-encoded virulence factors. As shown in Figure 7A, the expression of *ftnA* by the P*_lac_-ftn* gene on a plasmid greatly increased the level of EspB protein in the *fur* mutant, whereas the levels in the wild-type bacteria were not altered by the introduction of the P*_lac_-ftn* gene. The Fur protein can reduce the concentration of iron ion in the cytoplasm because Fur binds iron, and thus, we examined the effect of a Fur mutant protein lacking the ability to bind iron on *LEE* gene expression. The histidine residue in the iron-binding site [22] was replaced by isoleucine. The introduction of wild-type Fur into the *fur* mutant rescued the production of EspB, whereas the mutant Fur (FurH89I) could not increase the level of EspB in the *fur* mutant (Fig. 7, B). These results suggest that the intracellular concentration of free iron is a critical regulator of *LEE* gene expression. To further confirm the relationship between *LEE* gene expression and the intracellular free iron concentration, the overexpression and deletion of the *ryhB* gene were examined. A Fur-regulated sRNA, RyhB, regulates the expression of iron uptake genes and affects the intracellular iron concentration [23], [24]. Although the expression levels of EspB in the of *ryhB* deletion mutant were not altered compared with those in the wild-type bacteria, the deletion of the *ryhB* gene from the *fur* mutant increased the expression level of EspB (Fig. 7, C). Furthermore, the production of the MBP-Ler fusion protein from Ler66DWN was examined in this same set of strains. As shown for EspB, the amount of MBP-Ler in the *fur ryhB* double KO mutant was higher than that in the *fur* mutant (Fig. 7, D).

Using a streptonigrin sensitivity assay, we compared the intracellular concentrations of free iron in these strains. The growth of wild-type bacteria in DMEM containing 8 µg/ml streptonigrin was reduced to 87.9+/−0.4% of that without streptonigrin, but the growth of the *fur* mutant was more sensitive (reduced to 85.4+/−0.8%). The introduction of the *ryhB* mutation in the *fur* mutant rescued sensitivity, resulting in a level comparable to that in the wild type (Table 1). These results strongly suggest that the intracellular concentration of free iron is closely associated with the expression of *LEE* genes.

**Table 1:** Streptonigrin sensitivity.

Growth percentage against no addition		
	SNG	SNG
	(4µg/ml)	(8 µg/ml)
WT	90.6±0.4	87.9±0.4
*fur*	89.8±0.5	85.4±0.8
*ryhB*	90.3±0.6	88.1±0.7
*fur ryhB*	92.8±1.1	89.5±0.2

## Discussion

We found that the virulence genes of EHEC are regulated by the intracellular iron concentration. The identified regulatory mechanism targets the translation of the *ler* gene, which encodes the global virulence regulator Ler. Transcription of the antisense strand from a promoter downstream of *ler* is necessary for the bacteria to respond to changes in the intracellular concentration of free iron. Thus, we propose that the regulation of LEE and related virulence genes is closely linked to intracellular iron homeostasis.

The transcriptional regulator Fur plays a central role in bacterial iron homeostasis. The expression of genes related to iron uptake and utilization in EHEC is repressed by Fur during growth in medium containing high concentration of iron, such as LB, as shown for other bacteria. Although Fur affects the expression of *LEE* genes, the molecular mechanism of the Fur-regulated expression of *LEE* genes is different from the mechanism regulating Fur regulon genes. The expression of LEE-encoded virulence factors was greatly reduced by the deletion of the *fur* gene regardless of the concentration of iron in the medium. This result suggests that the mechanism of Fur-regulated *LEE* gene expression is not the same as that of genes belonging to the Fur regulon. The loss of the Fur regulator disrupts intracellular iron homeostasis through the deregulation of iron transport and utility systems. Previous reports have shown that the intracellular free iron concentration is elevated in *fur* mutants [24], [25]. We examined the expression of *LEE* genes under a variety of conditions that affect the intracellular free iron concentration. The overexpression of *ftnA*, which encodes the cytoplasmic iron-storage protein ferritin, increased the expression of EspB in the *fur* mutant. The introduction of a *ryhB* mutation into the *fur* mutant, which has been shown to reduce the iron concentration [24], rescued *espB* and *ler* expression. In addition, a streptonigrin sensitivity assay indicated that the free iron concentration in EHEC was higher in the *fur* mutant and that this level was reduced to a level comparable with that in wild-type bacteria by the additional introduction of a *ryhB* mutation. These results strongly suggest that increases in the intracellular free iron concentration reduce the expression of *LEE* genes. Furthermore, these results suggest that Fur contributes to the expression of *LEE* genes through an indirect mechanism. This hypothesis was further supported by the observation that gene expression could be rescued by the introduction of the *fur* gene on a plasmid into the *fur* mutant, whereas a plasmid expressing a mutant Fur that is unable to bind iron could not rescue gene expression. Thus, these results indicate that the expression of *LEE* genes is closely associated with intracellular free iron homeostasis.

The expression of *LEE* genes is regulated by the transcription factor Ler, which is encoded by the *ler* gene, the first gene in the *LEE1* operon. The *LEE1* operon promoter is activated by other transcription factors, including Pch proteins, which are encoded by phage-like genomes. Our search for the target of regulation in Fur-dependent expression revealed that a post-transcription step in *ler* expression was the point of regulation rather than the promoter activity of the *LEE1* operon. Although transcriptomic analysis showed that the transcription level of the *LEE1* operon was reduced in the *fur* mutant, the effect was not apparent for upstream genes of the operon. The transcript level of *ler* gene, which is the first gene in the *LEE1* operon, decreased only 19–34% with the introduction of the *fur* mutation. It is likely that the decrease in the transcript levels for downstream genes in the *LEE1* operon is caused by the degradation of transcripts rather than reduced transcription levels. Furthermore, the activity of the *LEE1* promoter in the *fur* mutant was almost the same as that in wild-type bacteria when measured using a luciferase fusion plasmid. These results suggest that transcription initiation from the *LEE1* promoter was not the target of the regulatory mechanism. This hypothesis was confirmed by the observation that the expression of *ler* from the P*lac-ler*1 plasmid, which contains the *ler* gene with its downstream sequence, was Fur dependent. Therefore, it is likely that interference with a post-transcriptional step of the expression of an upstream gene resulted in the degradation of the downstream transcripts of *LEE1*.

**Figure 8:**
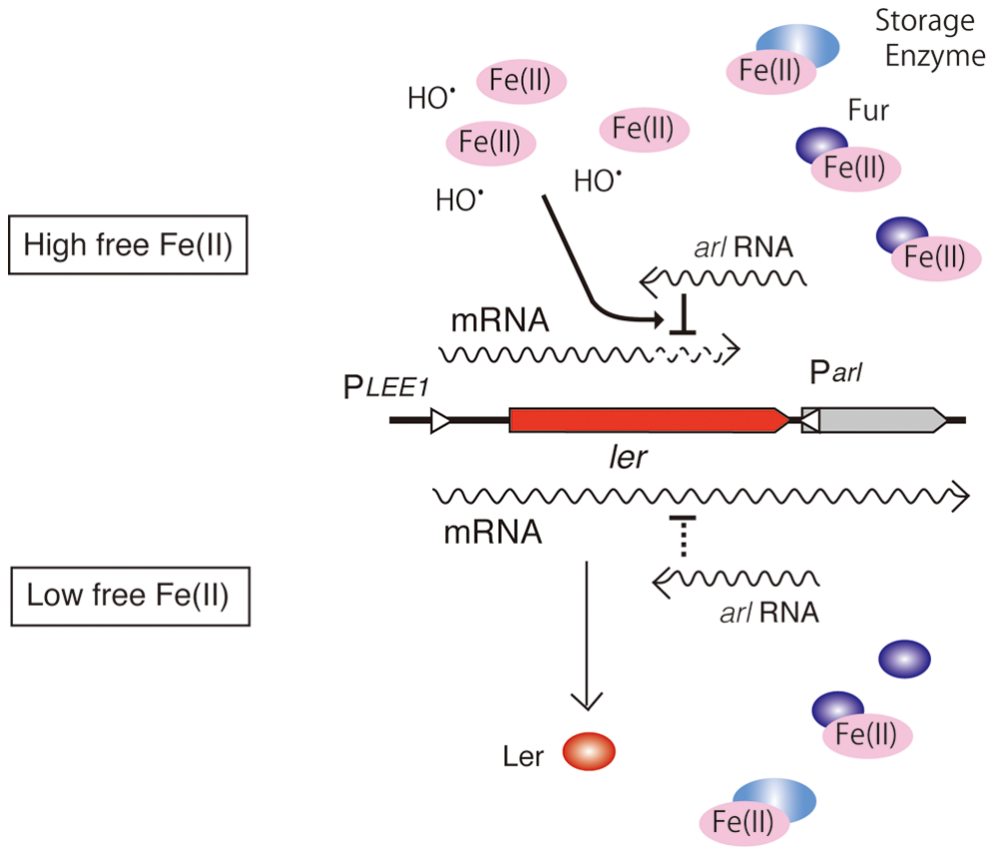
A schematic model of the iron-sensitive regulation of *ler* gene. Antisense RNA (*arl* RNA) is transcribed from downstream of *ler*. In the presence of high concentration of free iron in the cell, translation or stability of *ler* mRNA, transcribed from *LEE1* promoter, is reduced by the action of *arl* RNA, which is enhanced by iron or hydroxyl radical. While, in normal growth conditions, where intracellular free iron is low because iron is bound to iron-bound proteins such as storage enzymes, and Fur, inhibitory effect of *arl* RNA on *ler* expression is much reduced, resulting in the expression of *ler* and downstream genes.

Although expression of *ler* from a DNA fragment corresponding to only the coding sequence can overcome the effect of the *fur* mutation, the *ler* gene with the downstream sequence exhibited Fur-dependent expression. By constructing variety of MBP-Ler fusion genes, we showed that the translation of *ler* was necessary for the reduction in the *fur* mutant. Finally, we found that the opposite strand of the downstream sequence has promoter activity using luciferase fusion plasmids. The elimination of this promoter activity by deleting part of the downstream sequence resulted in the loss of Fur-dependent expression of MBP-Ler, indicating that the transcription of the opposite strand of the downstream sequence of the *ler* gene is necessary for the repression of *ler* translation in the *fur* mutant. Transcriptome analysis of the transcription start site in the EHEC O157 Sakai strain revealed transcription from 26 bp downstream of the *ler* stop on the antisense strand (manuscript in preparation). Interference with the translation of the C-terminal part of *ler* could be mediated by formation of a *ler* mRNA-antisense RNA complex, which is accelerated by a high concentration of free iron, or superoxide and hydroxyl radicals produced by free iron. Therefore, the antisense transcript, designated the *arl* RNA, plays a major role in the regulation of *ler* gene expression in response to changes in the intracellular iron concentration (Fig. 8). Non-coding RNAs have been identified in many bacteria, and some of them have been shown to be key regulators of gene expression [26]. Most of them are trans-encoded RNAs with partial antisense sequences complementary to target RNAs. In addition, recent comprehensive genome-wide analyses have revealed the presence of many cis-encoded antisense RNAs that are perfectly complementary to target RNAs [27]–[31]. The regulatory action of these RNAs can occur at the levels of transcription, mRNA stability or translation [32]. The expression of *ler* is regulated in response to disruptions of iron homeostasis through the antisense *arl* RNA. Our analysis showed that the translation of at least one-third of the *ler* mRNA is necessary and sufficient for the action of the antisense *arl* RNA. In addition, the introduction of a translational stop codon at junction of the MBP-ler fusion abrogated *fur*-regulated expression, and this effect was mediated by the *arl* RNA. Furthermore, the transcript level of *ler* in the *fur* mutant was lower than that in wild-type bacteria, as shown by transcriptome analysis. These results suggest that *arl* RNA affects both the stability of the RNA and the completion of *ler* translation. It is likely that the degradation of *ler* mRNA by formation of double-strand RNA with *arl* RNA in the downstream region results in the premature termination of translation. Thus, we propose a novel mechanism for *ler* expression in which the expression of this gene is regulated at the level of translation in response to the iron concentration through interference by a transcript from the antisense strand.

Sensing and response to changes in iron concentration could be important to enteric pathogens. Indeed, number of bacterial virulence factors has been shown to be regulated by the concentration of iron [6]. These virulence determinants include T3SS encoded by SPI1 of *Salmonella enterica*. Fur is involved in positive regulation of *hilA* gene, which encodes a master regulator of SPI1, through activation of *hilD*, encoding a positive regulator for *hilA* [33]. In *Salmonella*, Fur directly targets the *hilD* promoter and activates its transcription [34]. While, though expression of LEE encoding T3SS is repressed by high concentration of iron and Fur affect the response, the molecular mechanism implicated in the regulation of LEE in EHEC is dissimilar to that of SPI1 in *Salmonella*. Thus, the regulation at post-transcriptional level through the action of antisense RNA could be evolved as an alternative regulatory mechanism to respond to iron concentration.

## Materials and Methods

### Bacterial strains, media and growth conditions

The bacterial strains and plasmids used in this study are listed in Table 2. The deletion mutation for *fur* and *ryhB* were introduced into EHEC O157 Sakai using the method of Datsenko and Wanner [35]. Bacteria were grown in LB or DMEM with the addition of other reagents as described in the text. The pLux-P*_LEE1_* and pLux-P*_fepA_* plasmids were constructed by inserting a DNA fragment containing the *LEE1* promoter or the *fepA* promoter, which were isolated by PCR with primers LEE1-375-SalI and LEE1-D-NcoI and PfepA-U-Sal and PfepA-D-Bam, respectively, into pLux [36]. The pMAL-*ler* plasmid and its derivatives and the pLux-*ler*66DWN plasmid and its derivatives were constructed by inserting the DNA sequence of the *ler* gene together with the downstream sequence, which was isolated by PCR with appropriate primer set, into pMAL-c2x or pLux. The primers used for the isolation of specific DNA fragments are listed in Table 3.

**Table 2:** Bacterial strains and plasmids used in this study.

	Description	Reference
Strain		
Sakai (RIMD 0509952)	Wild type EHEC O157:H7	[39]
SKI1748	Sakai Δ*fur*	This study
SKI1163	Sakai pchA-FLAG DpchB DpchC	[19]
SKI1172	Sakai ler-FLAG	[19]
SKI0352	Sakai Δ*ler*	[17]
SKI1877	Sakai Δ*ler*Δ*fur*	This study
SKI1736	Sakai Δ*ryhB*	This study
SKI1801	Sakai Δ*fur* Δ*ryhB*	This study
Plasmid		
pLux	promoter-less lux operon	[36]
pLux-P*LEE1*	LEE1 promoter - lux operon fusion	This study
pLux-P*fepA*	fepA promoter-lux operon fusion	This study
pWKS-pchA-FLAG	Plac-pchA-FLAG	[19]
pTB101-pchA	Ptac-pchA	[19]
pWKS-ler-CD	Plac-ler coding sequence	This study
pWKS-ler1	Plac-ler with downstrem sequence	This study
pMAL-c2a	plasmid for MBP fusion construction	New England BioLabs
pMAL-ler11	MBP-Ler fusion without downstream sequence	This study
pMAL-ler11DWN	MBP-ler fusion with downstrem sequence	This study
pMAL-ler66DWN	deletion derivative of pMAL-ler11	This study
pMAL-ler96DWN	deletion derivative of pMAL-ler11	This study
pMAL-ler96DWN	deletion derivative of pMAL-ler11	This study
pMAL-lerDWN	MBP with only downstream sequence	This study
pMAL-ler66DWNOF	same as pMAL-ler66DWN but stop codon at junction	This study
pMAL-ler66U1	deletion derivative of pMAL-ler66DWN	This study
pMAL-ler66U2	deletion derivative of pMAL-ler66DWN	This study
pMAL-ler66U3	deletion derivative of pMAL-ler66DWN	This study
pGEM-ftnA	ftnA clone	This study
pGEM-fur	fur clone	This study
pGEM-fur(H89I)	fur(H89I) clone	This study
pLux-ler66DWN	opposite direction of ler-downstream sequence to lux operon	This study
pLux-ler66U1	deletion derivative of pLux-ler66DWN	This study
pLux-ler66U2	deletion derivative of pLux-ler66DWN	This study
pLux-ler66U3	deletion derivative of pLux-ler66DWN	This study

**Table 3:** Primers used for construction of plasmids.

LEE1-375-SalI	CTTCGTCGACGTGCTGGCTGTAGCTTATGTC
LEE1-D-NcoI	TGAGCCATGGGCTGTCGGCCTACGCCCGACCAGG
PfepA-D-Bam	TGTTGGATCCACCGCGAATATCAATCTGTCGG
PfepA-U-Sal	TGTAGTCGACAGACATCAGTACCTGCAATTCG
ler-N11-BamHI	TCCTGGATCCGAAAATAATTCACATACAACAAGTC
ler-N66-BamHI	TCCTGGATCCGTGCCTGATGATGGACTCGCTC
ler-N96-BamHI	GGAAGGATCCCAGCCACGCTGGCTTAAAGAAG
ECs4587-D-XhoI	GAACCTCGAGCTATTTATTATTAATCCTGATTCGCA
ler-STOP-BamHI	AGGTGGATCCTAACATGAAATAATTAAATGATAACGATAAC
asL1-U1	GTGTGTCGACGGTAACTTTATCCAAAGGGTG
asL1-U2	CTTGGTCGACCAATAAAAACATTTGCGGCTTC
asL1-U3	GTTGGTCGACATCTTCCAGCTCAGTTATCG

### Analysis of proteins in whole-cell lysates

Bacteria were collected from cultures by centrifugation, and the cell pellet was dissolved in SDS sample buffer. The concentration of each sample was normalized to the OD_600_ of the culture, and the samples were analyzed by immunoblotting after SDS-polyacrylamide (12% or 10%) gel electrophoresis (SDS-PAGE) and transfer to an Immobilon membrane (Millipore). The proteins were detected with primary antibodies specific for EspB, Tir [37], DnaK (Calbiochem) and FLAG (Sigma) and horseradish peroxidase-conjugated secondary antibodies, followed by visualization with an ECL detection kit (Amersham Biosciences).

### Promoter activity assay

Bacteria harboring promoter-luciferase fusion plasmids were grown in LB or DMEM after dilution (100-fold) of the overnight culture in LB. At the sampling time points, 800 µl was removed to measure the OD_600_, and 100 µl was taken to measure the luminescence intensity using a TD-20/20 luminometer (Turner Biosystems). The luciferase activity was calculated by dividing the luminescence intensity by the OD_600_. The average and standard error were calculated from the results of three experiments.

### 5′RACE

Total RNA was extracted and purified from EHEC strains with RNeasy Protect Mini Kit (Qiagen). Rapid amplification of cDNA ends (RACE) was performed with First-Choice RLM-RACE Kit (ambion) using manufacture's manual with some modifications. 5 µg of DNase I-treated RNA was treated with TAP (Tabacco Acid Pyrophosphatase) or left untreated, and then 5′ RACE Adapter was ligated. cDNA was synthesized from Adapter-attached RNA with random decamer. 5′-end of *arl* was amplified by PCR with primers (5′ RACE Outer Primer and ler-N66-BamHI), and products were visualized after electrophoresis in Gel-Red containing agarose gel.

### Streptonigrin sensitivity assay

The streptonigrin sensitivity of each strain was measured by comparing the growth in DMEM with or without streptonigrin, as previously described [38]. Briefly, bacteria were collected from overnight cultures in LB, washed with DMEM, and then resuspended in DMEM. DMEM containing DMF (dimethyl formamide) or streptonigrin was inoculated using a bacterial suspension (to be OD_600_ = 0.1) and incubated at 37°C with shaking for 18 h. Growth was measured by the OD_600_, and the percentage inhibition of growth was calculated with respect to the growth of corresponding control cultures. The average percentage and standard error were determined from the results of three experiments.

### Transcriptome analysis

Total RNA from EHEC cells was isolated with an RNeasy kit (QIAGEN) using the method recommended by the manufacturer. The RNA was further purified after treatment with RNase-free DNase I (Takara). The Affymetrix GeneChip *E. coli* Genome 2.0 array was used to compare the transcriptomes of wild-type and *fur* mutant EHEC O157 Sakai. The processing of the extracted RNA, cDNA labeling, hybridization and slide scanning were performed according to manufacturer's instructions (http://www.affymetrix.com). Two independent experiments for each condition were performed. The data corresponding to EHEC O157 Sakai were extracted and normalized based on the total signal intensity. The ratio of the transcript levels for each gene was calculated as an average of the ratios for four combinations of two experiments for each condition.
